# Evidence of Iron Accumulation in Cerebral Adrenoleukodystrophy: A Potential Novel Disease Mechanism

**DOI:** 10.1002/acn3.70346

**Published:** 2026-02-24

**Authors:** Christina L. Nemeth, Mert Sisman, Jinwei Zhang, Hyeong‐geol Shin, Xu Li, Bela Turk, Ali Fatemi, Thanh Nguyen, Eric Mallack

**Affiliations:** ^1^ Moser Center for Leukodystrophies, Kennedy Krieger Institute Baltimore Maryland USA; ^2^ Department of Neurology The Johns Hopkins University School of Medicine Baltimore Maryland USA; ^3^ Department of Radiology Weill Cornell Medicine New York City New York USA; ^4^ Department of Electrical and Computer Engineering Cornell University Ithaca New York USA; ^5^ Department of Electrical and Computer Engineering Johns Hopkins University Baltimore Maryland USA; ^6^ The F.M. Kirby Research Center for Functional Brain Imaging Baltimore Maryland USA; ^7^ Department of Radiology The Johns Hopkins Hospital Baltimore Maryland USA

**Keywords:** adrenoleukodystrophy, ferroptosis, iron, lipid peroxidation, quantitative susceptibility mapping, source separation

## Abstract

In this first application of Quantitative Susceptibility Mapping Source Separation to cerebral adrenoleukodystrophy, we uncovered alterations in iron and myelin within lesions and normal appearing white matter. As validation, we demonstrate abnormal iron accumulation in those same compartments within primary brain tissue. A gradient of microglial activation to absence parallels the transition from normal white matter to demyelinated lesion. In perilesional white matter, increases in myelin peroxidation and Acyl‐CoA synthetase long‐chain family member 4 are observed. Thus, the unifying mechanistic relationship of apoptosis to lipid peroxidation in the presence of iron implicates ferroptosis in the pathogenesis of cerebral adrenoleukodystrophy.

**Abbreviations**
ACSL4: acyl‐CoA synthetase long‐chain family member 4
CALD: cerebral adrenoleukodystrophy
NAWM: normal appearing white matter
QSM: quantitative susceptibility mapping

## 1 Introduction

X‐linked adrenoleukodystrophy (ALD) is a neurodegenerative disorder caused by impaired very long‐chain fatty acid (VLCFA) metabolism. VLCFAs incorporate and compromise cell membrane function in intact white matter [1]. Intercalation into the inner mitochondrial membrane disrupts oxidative phosphorylation, creating redox imbalance, an impaired stress response, and lipid peroxidation [2].

At the cellular level, a defective microglial response and subsequent apoptosis precede the spatial progression of cerebral ALD (CALD), the ultimately fatal, inflammatory demyelinating phenotype [3]. Significant challenges are associated with a diagnosis of CALD [4, 5, 6, 7, 8, 9] and early prognostic biomarkers remain elusive. Identifying mechanisms that precede lesion formation is critical.

We employed Quantitative Susceptibility Mapping (QSM) Source Separation for the first time in CALD. QSM decodes the distribution of tissue magnetic susceptibility—a property characterized by perturbations in the tissue's local magnetic field induced by an external field (e.g., MRI scanner). The overall tissue susceptibility signal can be “source‐separated” into component maps of paramagnetic molecules (sensitive to iron), which enhance (paramagnetism:QSM positive:QSMp), and diamagnetic molecules (sensitive to myelin), which repel (diamagnetism:QSM negative:QSMn), the external field [10]. An increase in paramagnetic susceptibility due to brain iron overload has been mechanistically linked to ferroptosis [11]. Reduction in the diamagnetic contribution of myelin may result from membrane lipid peroxidation‐mediated myelin breakdown [12], and has been demonstrated in multiple sclerosis [13]. Source separation has differentiated lesions across multiple neurological pathologies [14].

Informed by our QSM findings, we performed an immunohistochemical analysis to evaluate for the presence of iron and to measure two early pathological events acting in concert—lipid peroxidation and microglial apoptosis. We hypothesized that abnormal iron accumulation detected on QSMp would be demonstrable in brain tissue, and that a decrease in the diamagnetic signal beyond the lesion would colocalize with lipid peroxidation and microglial loss. Given the unifying mechanistic relationship of apoptosis to lipid peroxidation, we suspect that ferroptosis is a central mechanism driving CALD.

## 2 Methods

### 2.1 Quantitative Susceptibility Mapping Source Separation

We analyzed MR datasets from an 8‐year‐old with progressive CALD (enhancing lesion on T1 post‐contrast MRI, progressed over 1 year and 2 months), and a 6‐year‐old with arrested CALD (no lesional enhancement, self‐stabilized for 2 years and 8 months). Patients were scanned on the same 3 T MRI scanner under the same “Leukodystrophy Protocol.” Fifty‐two control datasets from boys aged 5–10 years were acquired during routine clinical care (MRI acquisition protocols are detailed in Supporting Information S1). The QSM acquisition protocol was harmonized and demonstrated to be reproducible across manufacturers [15, 16].

QSM for all study subjects underwent the same processing pipeline (QSM source separation pipeline is detailed in Supporting Information S2). QSMp (paramagnetic) and QSMn (diamagnetic) control atlases were created. ALD maps were compared to the normative atlases. Variation was quantified by voxel‐wise *Z*‐score; increases in paramagnetism or diamagnetism are red, decreases are blue. A coordinate, quantitative region of interest analysis across the T2 FLAIR sequence, T1 post‐contrast sequence, Raw source‐separated QSM, and QSM *Z*‐Score map was performed.

### 2.2 Immunohistochemical Analysis of Primary Brain Tissue

Immunohistochemical analysis of primary brain tissue (NIH NeuroBioBank) was performed in one control (69 years old) and three patients with CALD (25, 17, and 10 years old). Formalin‐fixed tissue from Broca's area 40 (BA40) and periventricular white matter was cryostat sectioned at 20 μm and stained for Acyl‐CoA synthetase long‐chain family member 4 (ACSL4, Proteintech; 1:500), an enhancer of lipoxidation requisite for the ferroptosis pathway [17], and 4‐Hydroxynonenal (4HNE), a marker of lipid peroxidation, in normal appearing cortex and white matter, and in areas of demyelination. Quantification and colocalization of these proteins were done within the context of iron deposition (Abcam Iron Stain Kit; ab150674), oligodendrocytes (MBP, CC1) and microglia (IBA1). Primary incubation was performed overnight, followed by 1 h secondary exposure (1:500), and coverslipping with Fluoromount. Myelin status was confirmed using Luxol Fast Blue (LFB) overnight at 57°C and counterstained with cresyl violet. Tissue was imaged on a Leica DMi8 Thunder Imager with computational clearing.

### 2.3 Institutional Approvals and Patient Consent

Imaging studies were acquired during clinical care and analyzed under an institutionally‐approved research protocol at Weill Cornell Medicine with waiver of consent due to anonymization. Post‐mortem tissue is anonymized and available through the NIH NeuroBioBank.

## 3 Results

### 3.1 Pathological Alterations in Iron and Myelin Signals on Source‐Separated QSM

**FIGURE 1:**
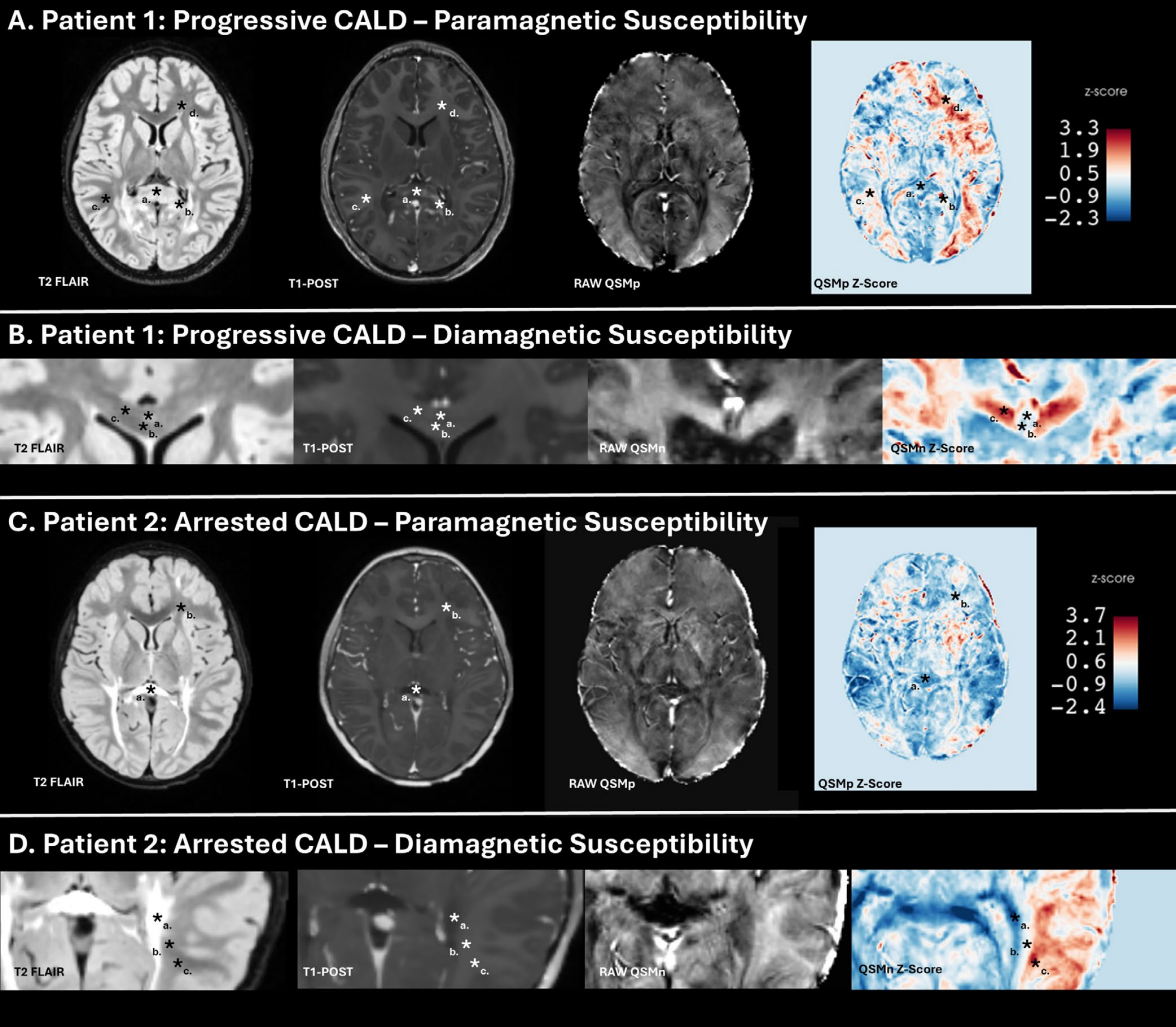
Pathological alterations in iron and myelin signals on source‐separated QSM in CALD. A detailed region of interest analysis across the T2 FLAIR sequence, T1‐post contrast sequence, Raw source‐separated QSM, and QSM *Z*‐score map for Patients 1 and 2. (A) Paramagnetic susceptibility on QSMp in Patient 1 is (a.) decreased in the non‐enhancing area of demyelination, (b.) elevated in the area of enhancement at the leading edge of the lesion, and elevated in (c.) perilesional NAWM and (d.) remote NAWM. (B) Diamagnetic susceptibility on QSMn in Patient 1 is reduced in (a.) corpus callosum lesion and (b.) penumbra of immediate perilesional NAWM, and (c.) increased in distal perilesional NAWM. (C) Paramagnetic susceptibility on QSMp in Patient 2 is decreased in (a.) corpus callosum lesion and (b.) remote NAWM. (D) Diamagnetic susceptibility on QSMn in Patient 2 is (a.) reduced in periventricular white matter lesion, (b.) normalizes in immediate perilesional NAWM, and (c.) increased in distal perilesional NAWM. Corresponding values per coordinate annotation in Figure 1 are provided in Table 1.

Our analysis revealed pathological alterations in QSMp and QSMn maps within cerebral lesions and in areas of the brain that appear normal. The quantitative region of interest analysis corresponding to the coordinate annotations in Figure 1 is provided in Table 1. Both patients demonstrated a gradient of decreased to increased paramagnetic and diamagnetic susceptibilities across the T2 lesion to NAWM, quantified by region of interest *Z*‐score in Table 1.

**TABLE 1:** Quantitative Region of Interest Analysis Corresponding to Figure 1.

Patient ID	Phenotype	Source separated map	Annotation Figure 1	Coordinate brain region of interest measurement	T2 FLAIR raw signal intensity	T1‐Post raw signal intensity	QSM raw value (ppb)	QSM *Z*‐score vs. control atlas
Patient 1	Progressive CALD	Row A: QSMp (paramagnetic)	(a.)	Lesion: midline splenium corpus callosum, no T1‐post contrast enhancement	3023	1597	9.9	−1.7
			(b.)	Lesion: left paramedian splenium corpus callosum, T1‐post contrast enhancement	2615	2994	51.0	3.7
			(c.)	Perilesional NAWM: right parietal subcortical	1404	2290	42.2	1.1
			(d.)	Remote NAWM: Left Frontal Subcortical	1900	1749	39.8	2.3
		Row B: QSMn (diamagnetic)	(not shown)	Lesion: midline splenium corpus callosum, no T1‐post contrast enhancement	3281	1088	4.8	−2.7
			(a.)	Lesion: midline genu corpus callosum, no T1‐post‐contrast enhancement	2651	1740	19.6	−0.2
			(b.)	Immediate perilesional NAWM: genu corpus callosum	1790	1883	5.5	−0.8
			(c.)	Distal perilesional NAWM: genu corpus callosum	1819	2022	47.8	2.9
Patient 2	Arrested CALD	Row C: QSMp (paramagnetic)	(a.)	Lesion: midline splenium corpus callosum, no T1‐post contrast enhancement	3447	585	10.0	−0.7
			(b.)	Remote NAWM: left frontal subcortical	1904	1200	26.0	−0.4
		Row D: QSMn (diamagnetic)	(a.)	Lesion: left periventricular white matter	3718	973	16.9	−2.3
			(b.)	Immediate perilesional NAWM: left occipital subcortical	1776	1311	36.0	0.6
			(c.)	Distal perilesional NAWM: left occipital subcortical	1591	1326	42	3.9

Abbreviation: Ppb, parts per billion.

Specific to Patient 1, there are elevated paramagnetic signals in the corpus callosum colocalized to the leading edge of inflammatory demyelination (Figure 1A, annotation b.; Table 1, Row A, annotation b.) and the remote NAWM of the left hemisphere (Figure 1A, annotation d.; Table 1, Row A, annotation d). On the QSMn map, there is a reduction of the diamagnetic signal not only within the T2 lesion, but in a penumbra of the immediate perilesional NAWM (Figure 1B, annotation b.; Table 1, Row B, annotation b).

Contrary to Patient 1, Patient 2 demonstrated an overall lower volume of paramagnetic (Figure 1A,C) and diamagnetic elevations across the whole brain, indicating that disease stability may correlate with less severe molecular and cellular pathologies driving the observed alterations in source‐separated magnetic susceptibility.

### 3.2 Iron Accumulation in Demyelinated and Normal Appearing White Matter

**FIGURE 2:**
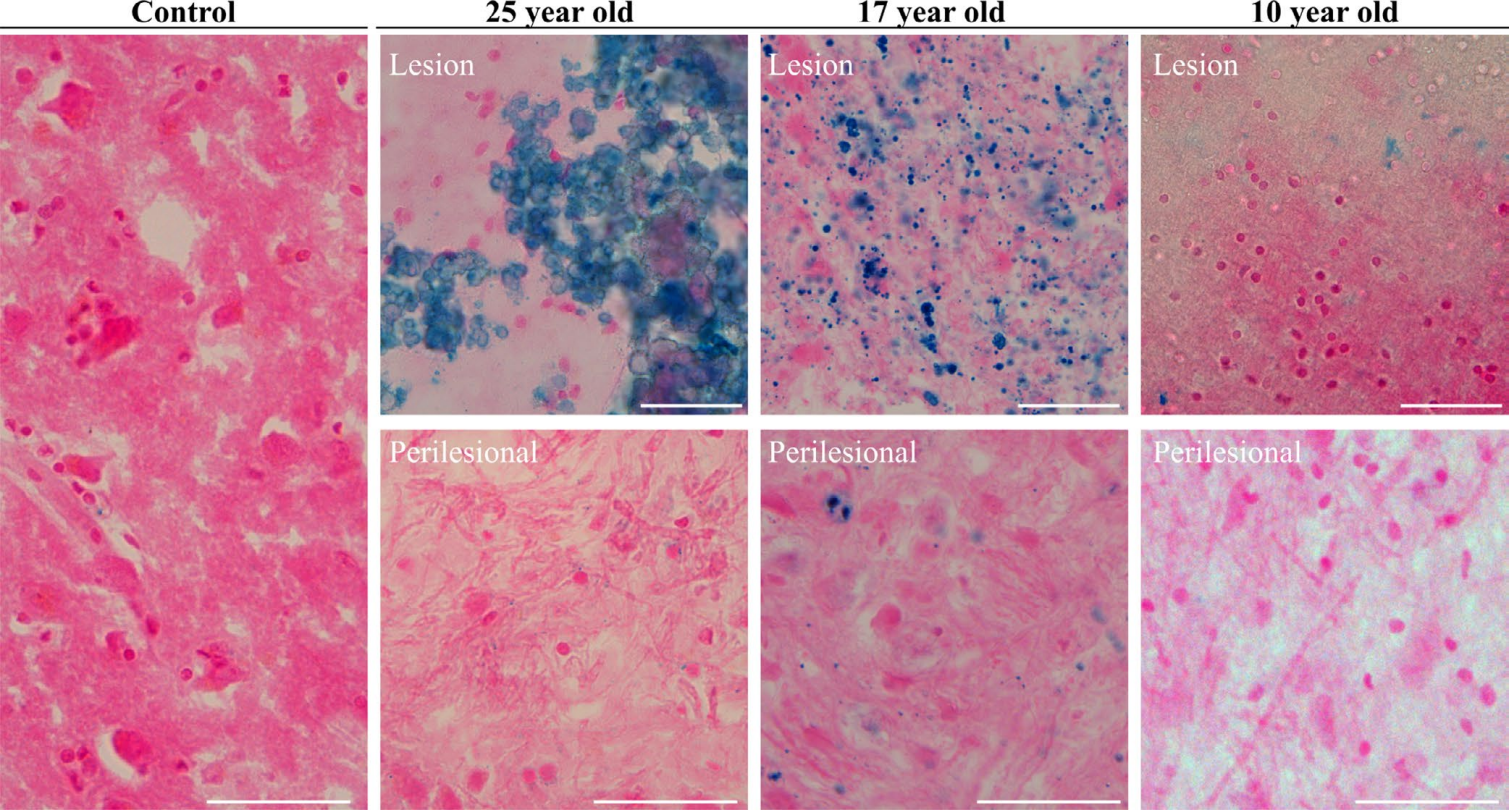
Abnormal iron accumulation in the CALD brain. Prussian Blue staining of a 69yo control versus ALD postmortem tissue showing significant iron accumulation is present within a CALD lesion and is diffusely present in perilesional WM in 10‐, 17‐, and 25‐year‐old patients. All scale bars indicate 50 μm.

Primary brain tissue was assessed with Prussian blue staining for ferric iron in one control sample and three CALD patient samples. Staining revealed differential labeling across the transition from perilesional NAWM to lesion, which corresponds to loss of LFB, likely reflective of each patient's degree of disease‐related injury. Versus control (Figure 2, column 1), the 25‐year‐old and 17‐year‐old patients' samples overall show diffuse iron in perilesional areas with disorganized fibers beyond the leading edge of the lesion. Large deposits of iron are noted within the demyelinating lesion of the 25‐year‐old. Similarly, a significant increase in diffuse iron staining with variable clumping of iron is present within the demyelinating lesion of the 17‐year‐old patient (Figure 2, columns 2–3).

The 10‐year‐old patient shows a greater severity of disease; the periventricular WM was necrotic, as defined nuclear fast red signal that was largely absent. Deposits of iron are noted within demyelinated areas. Within the perilesional region, brain iron was faint and not well defined, consistent with iron release following cell death into extracellular spaces (Figure 2, column 4).

### 3.3 Molecular Markers of the Ferroptosis Pathway Parallel Transition From Normal Appearing White Matter to Demyelination

**FIGURE 3:**
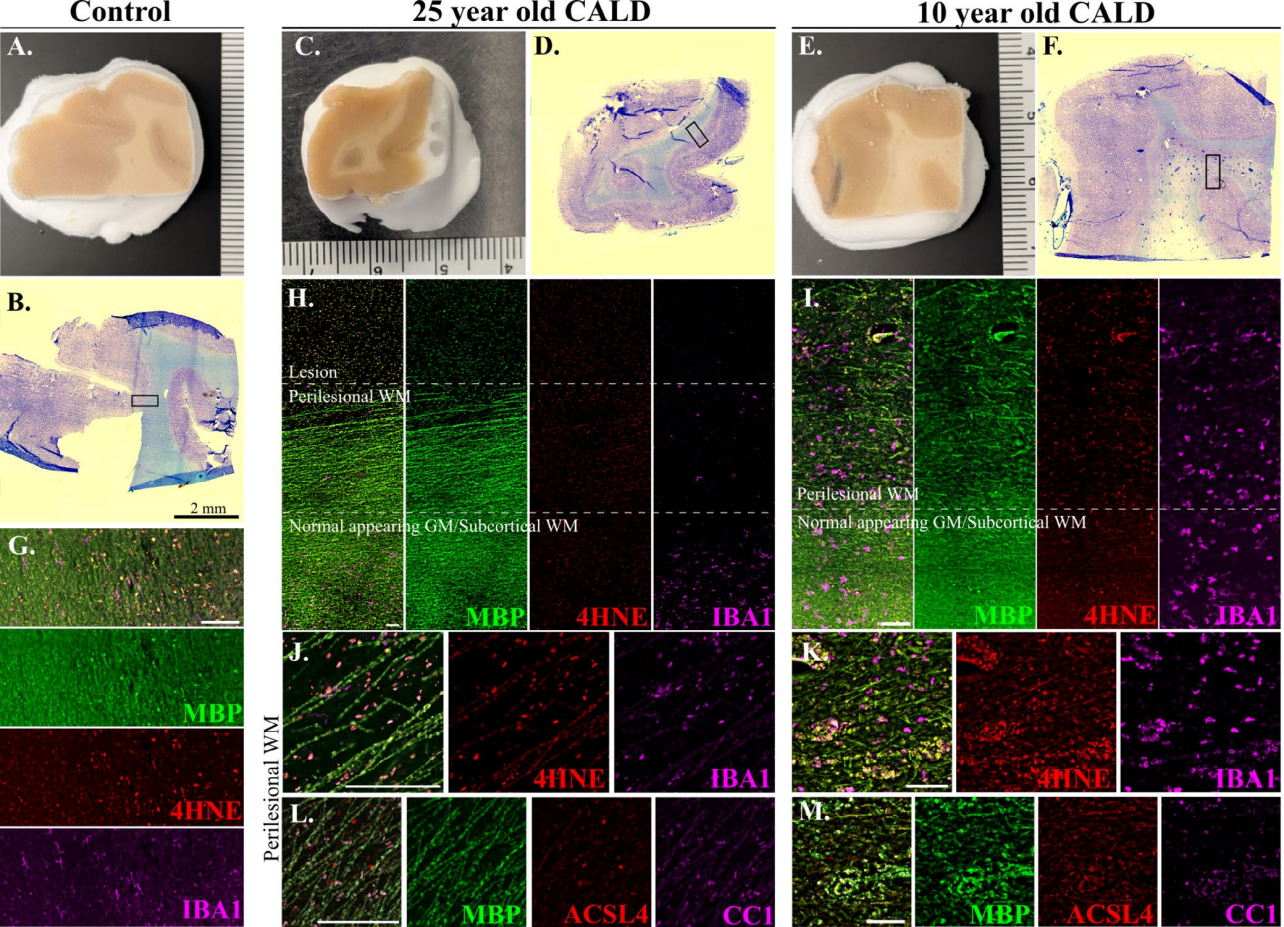
Markers of ferroptosis parallel the gradient of normal appearing white matter to lesion. Primary brain tissue from Broca's area 40 (inferior parietal lobe) from a 69yo control patient, 25yo and 10yo CALD patients to measure markers of ferroptosis and lipid peroxidation in normal appearing and demyelinated tissue. (A,B) Uniform LFB staining across control tissue (rectangle in B) is in contrast to (C–F) heterogeneous staining within two CALD samples. (G) Higher magnification of control brain staining for myelin (MBP), lipid peroxidation (4HNE), and microglia (IBA1) reveals normal signals across the cortex to subcortical NAWM with ramified microglial morphology. (H–K): Higher magnification imaging of CALD tissues across a lesion (rectangles in D and F) indicated by changing LFB intensity are shown with MBP, 4HNE, and IBA1 populations. Normal appearing myelin shows dense MBP staining, which transitions to perilesional spaces marked by areas of reduced myelin compaction and organization, and axonal swelling, which progresses to lesioned areas denoted by cell debris, fragmentation, and loss of cell identification. Ameboid morphology to loss of IBA1 signal parallels the transition from NAWM to demyelination. (J, K) Evidence of increased lipid peroxidation (4HNE) colocalized with myelin (MBP) and microglia (IBA1) in perilesional WM. (L, M) Acyl‐CoA synthetase long‐chain family member 4 (ACSL4), a pro‐ferroptotic regulator, is present and partially colocalizes with myelin (MBP, CC1) in perilesional WM. All scale bars indicate 100 μm.

BA40 was assessed in control and two CALD patients' postmortem brain tissue (25‐year‐old and 10‐year‐old patients) to measure markers of ferroptosis and lipid peroxidation within the lesion and normal appearing tissue. Uniform LFB staining across control tissue is in contrast to heterogeneous staining within CALD samples as compared to white matter within matched images of bulk tissue (Figure 3A–F).

Higher magnification imaging within these regions of changing LFB intensity are shown with myelin (MBP), lipid peroxidation (4HNE), and microglial populations (IBA1). Across both patient samples, normal appearing myelin (characterized by LFB) shows dense MBP staining, which transitions to perilesional spaces marked by areas of reduced myelin compaction and organization, and axonal swelling, which progresses to lesioned areas denoted by cell debris, fragmentation, and loss of cell identification. Ramified to ameboid to loss of IBA1 signal parallels transition from NAWM to demyelination, validating prior work demonstrating activated microglia in extralesional NAWM transitioning to microglial apoptosis in perilesional and lesional tissue (Figure 3H,I) [3]. Myelin and microglial markers within perilesional spaces colocalize with the presence of 4HNE (Figure 3H–K), while ACSL4 is largely present outside of MBP+ myelin (Figure 3L,M) and likely varies across disease states and tissues.

## 4 Discussion

To our knowledge, this is the first application of QSM in CALD. This study demonstrates the first evidence of abnormal iron content in primary brain tissue detectable by imaging. Our findings suggest that elevated paramagnetic susceptibility in normal appearing white matter may correlate with increased iron uptake in activated microglia [18], and reduction in the diamagnetic signal spatially corresponds to the presence of myelin lipid peroxidation [12] beyond the leading edge of the lesion [19].

The second novel contribution is the gradient of lipid peroxidation and expression of ACSL4, which parallels the transition of microglial activation to loss in primary brain tissue. Lipid peroxidation is the final effector of iron‐dependent cell death [20] facilitated by ACSL4‐mediated cell membrane enrichment of long polyunsaturated fatty acids essential for execution of the ferroptosis pathway [17]. Given the unifying mechanistic relationship of apoptosis to lipid peroxidation in the presence of iron [18], this exploratory study provides the first evidence that ferroptosis may play an early role in the pathogenesis of CALD.

Several limitations exist: generalizability is limited by small sample size; variability and registration in the normative atlas may introduce bias; and histological findings represent end‐stage tissue changes that may not fully capture dynamic disease processes.

This is a novel avenue of research in Adrenoleukodystrophy. Cell‐type‐specific expression of ferroptotic markers suggests a contribution of this pathway to disease pathogenesis. Source‐separated QSM may identify those at risk for developing CALD and inform progression versus stabilization. Further validation of the QSM signals against histology and mechanistic interrogation of the cellular and molecular findings has the potential to confirm ferroptosis as a disease mechanism in CALD.

## Author Contributions

**Christina L. Nemeth:** conceptualization; investigation; writing – original draft; methodology; visualization; formal analysis; data curation; writing – review and editing. **Mert Sisman:** conceptualization; investigation; methodology; visualization; formal analysis; data curation; writing – review and editing. **Jinwei Zhang:** methodology; writing – review and editing; investigation. **Hyeong‐geol Shin:** investigation; methodology; writing – review and editing; formal analysis. **Xu Li:** investigation; methodology; writing – review and editing. **Bela Turk:** investigation; methodology; writing – review and editing. **Ali Fatemi:** funding acquisition; supervision; resources; writing – review and editing; visualization; methodology. **Thanh Nguyen:** conceptualization; investigation; methodology; visualization; formal analysis; resources; writing – review and editing. **Eric Mallack:** conceptualization; funding acquisition; investigation; writing – original draft; methodology; visualization; writing – review and editing; formal analysis; project administration; data curation; supervision; resources.

## Funding

This work was supported by Eunice Kennedy Shriver National Institute of Child Health and Human Development, P50HD103538, National Institute of Neurological Disorders and Stroke, K23NS118044.

## Conflicts of Interest

The authors declare no conflicts of interest.

## Supporting information


**Data S1**: MRI Acquisition Protocol.

**Data S2**: QSM Source Separation Pipeline.

## Data Availability

After publication, any data not published within this article will be anonymized and shared upon reasonable request from a qualified investigator.
