# Supplementary material for: Evidence of Iron Accumulation in Cerebral Adrenoleukodystrophy: A Potential Novel Disease Mechanism
Source: Ann Clin Transl Neurol. 2026 Feb 24;13(6):1276–82. doi: 10.1002/acn3.70346 (PMC13251430; doi:10.1002/acn3.70346)
Supplement: Supplementary file 1 — Data S1: MRI Acquisition Protocol. [file ACN3-13-1276-s001.docx]

**Supplement 1: MRI Acquisition Protocol**

ALD Patients:

ALD Patient 1 and 2 were scanned on the same MRI Scanner under the same clinical “Leukodystrophy MRI Protocol” which included 3D-TI, 3D-T2, 3D-T2 FLAIR for anatomic structure, gadolinium-enhanced 3D-T1 to detect blood-brain-barrier disruption, and QSM.

Model: GE Discovery 3T MR 750W, 24 channel headcoil

QSM: Scanning Sequence = RM (gradient echo-based sequence), Slice Thickness = 3.00mm, Acquisition Matrix 416 x 320, Image Matrix 512 x 512, TR = 48.407ms, TE = 38.516ms, Echo Train Length = 6, Echo Spacing = 6.6ms, Bandwidth = 244 Hz/Px, Flip Angle = 20°, Number of Averages = 0.696121, Slice Spacing = 3.00mm. Resulting voxel size = 0.4297mm x 0.4297 mm x 1.00mm.

Control Patients:

Brain MRIs were performed on 3T MR imaging scanners during routine clinical care. The scanning protocols consisted of standard 3D-T1, 2D-T2, and 3D-T2 FLAIR sequences for anatomic structure, and gadolinium-enhanced 3D-T1 to detect blood-brain barrier disruption, and QSM.

Models: GE Discovery 3T MR 750W, 24 channel headcoil; GE Signa HDxt, product 8-channel head coil; Siemens Magnetom Skyra, product 20-channel head/neck coil.

QSM: Scanning sequence = multiecho 3D gradient recalled-echo (GRE) sequence, Slice Thickness = 3.00 mm, Acquisition Matrix 320 – 416 x 205 – 320 , FOV = 240mm x 240mm, TR = 49 – 58ms, TE = 47.7ms, Echo Train Length = 8 – 10, Echo Spacing = 4.1 – 4.8ms, Bandwidth = 244 – 260 Hz/pixel, Flip Angle 15°– 20°, Number of Averages = 1.

Reproducibility:

The QSM acquisition protocol was harmonized for scanner manufacturers and was demonstrated to be reproducible across manufacturers.^1,2^

**REFERENCES**

1. Deh, K. *et al.* Reproducibility of quantitative susceptibility mapping in the brain at two field strengths from two vendors. *Journal of Magnetic Resonance Imaging* 42, 1592–1600 (2015).

2. Deh, K. *et al.* Multicenter reproducibility of quantitative susceptibility mapping in a gadolinium phantom using MEDI+0 automatic zero referencing. *Magn Reson Med* 81, 1229–1236 (2019).
