# Supplementary material for: Evidence of Iron Accumulation in Cerebral Adrenoleukodystrophy: A Potential Novel Disease Mechanism
Source: Ann Clin Transl Neurol. 2026 Feb 24;13(6):1276–82. doi: 10.1002/acn3.70346 (PMC13251430; doi:10.1002/acn3.70346)
Supplement: Supplementary file 2 — Data S2: QSM Source Separation Pipeline. [file ACN3-13-1276-s002.docx]

**Supplement 2: QSM Source Separation Pipeline**

QSM was reconstructed from complex multi-echo 3D gradient echo images using a fully automated Morphology Enabled Dipole Inversion algorithm zero-referenced to the ventricular cerebrospinal fluid.^1,2^ Maps were generated through nonlinear field fitting^3^ and background field removal,^4^ then processed using maximum spherical mean value filtering.^5^ R2* maps were generated via auto-regression on linear operators.^6^ Source separation was carried out using R2*-based approach **(Supplement 2, Figure 1).**^7^ The magnitude atlas was normalized and registered using Advanced Normalization Tools^8^ onto the NIHPD pediatric atlas.^9^ Sex-specific QSMp (paramagnetic) and QSMn (diamagnetic) control atlases were created using data from 52 age-matched male subjects aged 5-10 years **(Supplement 2, Figure 2)**. QSMn and QSMp maps from the two ALD patients were compared to the normative male atlases in 3-D slicer.^10,11^ Variation in ALD maps versus the control atlas were quantified by voxel-wise Z-score; voxel-wise increases in paramagnetism or diamagnetism are red, decreases are blue.


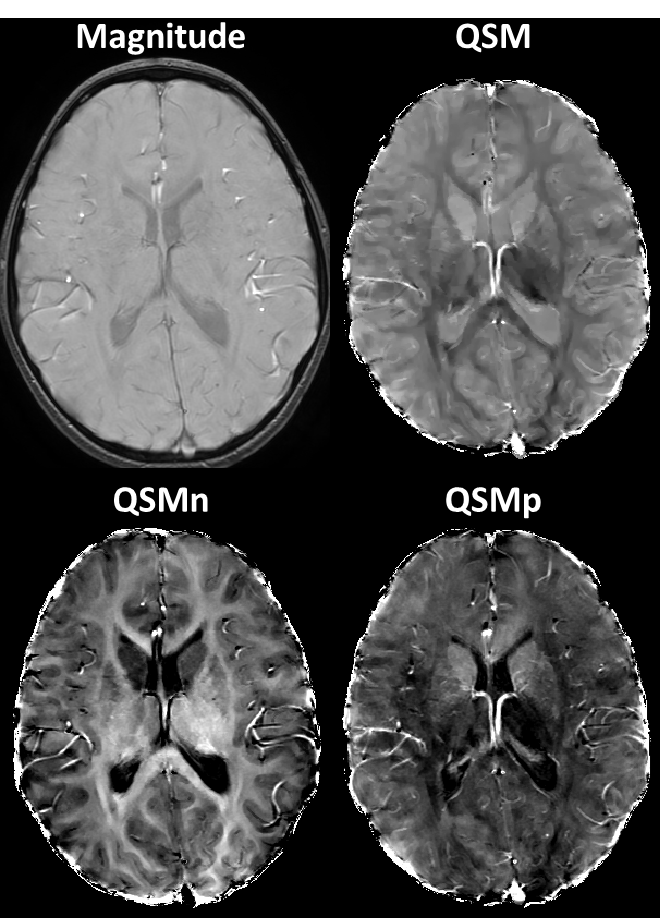


**Supplement 2, Figure 1:** Single case example of the Magnitude, QSM, and source-separated QSMn (diamagnetic) and QSMp (paramagnetic) maps.


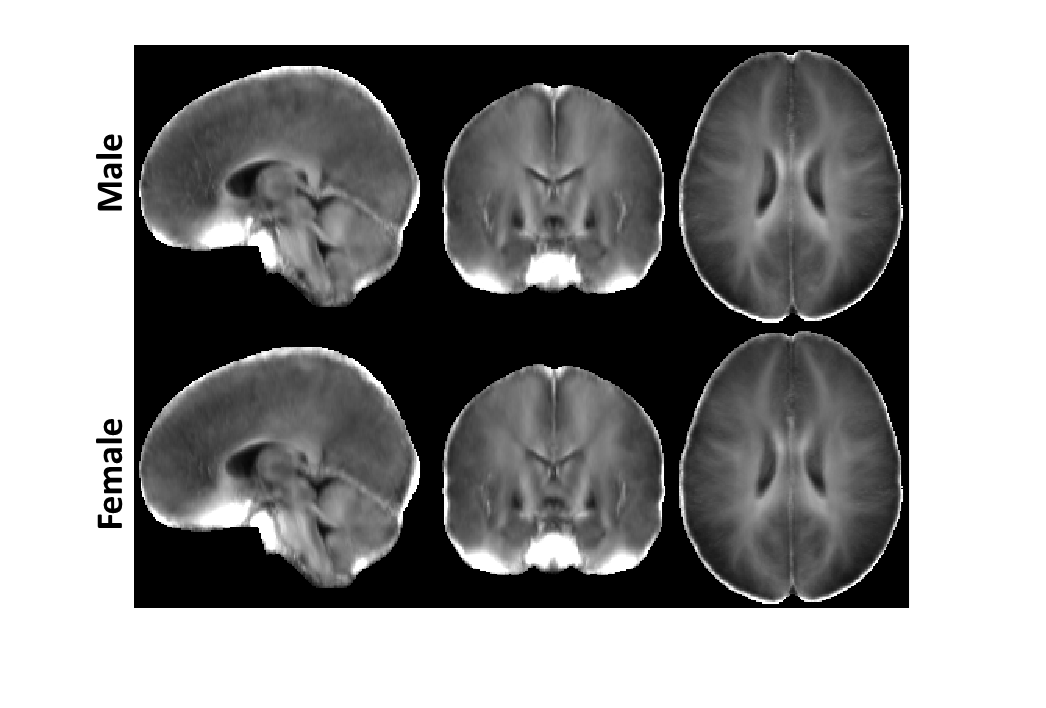

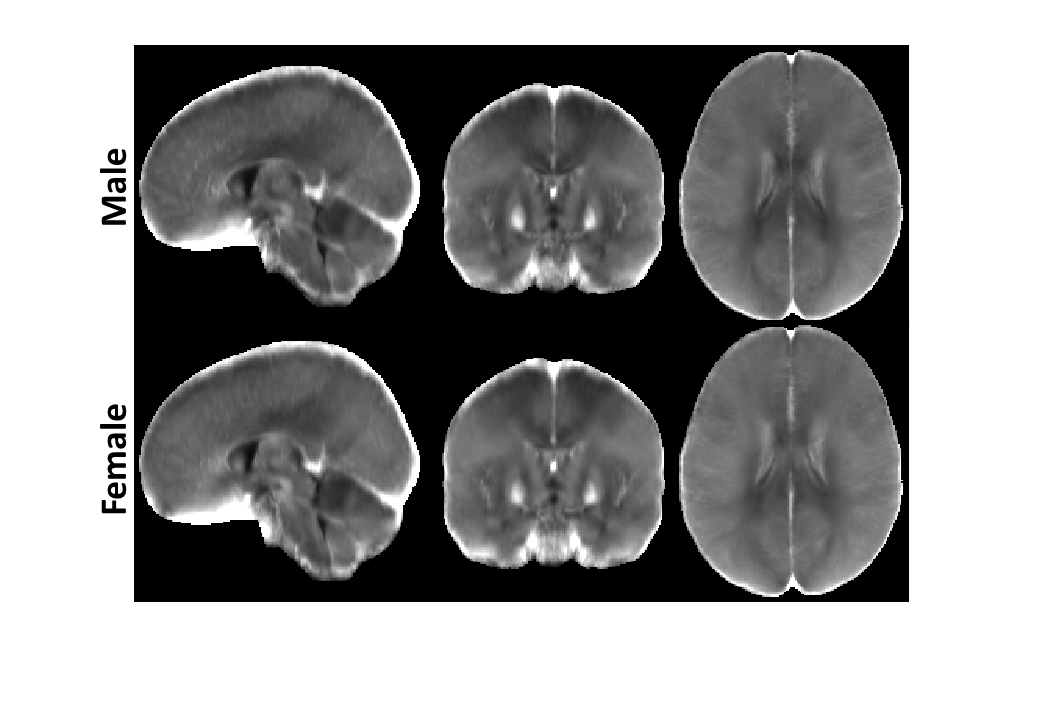


**B**

**A**

**Supplement 2, Figure 2:** (A) QSMn (diamagetic) control atlas for age-matched males (top row) and females (bottom row). (B) QSMp (paramagetic) control atlas for age-matched males (top row) and females (bottom row).

**REFERENCES**

1. Liu, Z., Spincemaille, P., Yao, Y., Zhang, Y. & Wang, Y. MEDI+0: Morphology enabled dipole inversion with automatic uniform cerebrospinal fluid zero reference for quantitative susceptibility mapping. *Magn Reson Med* 79, 2795–2803 (2018).

2. De Rochefort, L. *et al.* Quantitative susceptibility map reconstruction from MR phase data using bayesian regularization: Validation and application to brain imaging. *Magn Reson Med* 63, 194–206 (2010).

3. Liu, T. *et al.* Nonlinear formulation of the magnetic field to source relationship for robust quantitative susceptibility mapping. *Magn Reson Med* 69, 467–476 (2013).

4. Liu, T. *et al.* A novel background field removal method for MRI using projection onto dipole fields (PDF). *NMR Biomed* 24, 1129–1136 (2011).

5. Roberts, A. G. *et al.* Maximum spherical mean value filtering for whole-brain QSM. *Magn Reson Med* 91, 1586–1597 (2024).

6. Pei, M. *et al.* Algorithm for fast monoexponential fitting based on Auto-Regression on Linear Operations (ARLO) of data. *Magn Reson Med* 73, 843–850 (2015).

7. Dimov, A. V. *et al.* Susceptibility source separation from gradient echo data using magnitude decay modeling. *Journal of Neuroimaging* 32, 852–859 (2022).

8. Avants, B. B. *et al.* A reproducible evaluation of ANTs similarity metric performance in brain image registration. *Neuroimage* 54, 2033–2044 (2011).

9. Fonov, V. *et al.* Unbiased average age-appropriate atlases for pediatric studies. *Neuroimage* 54, 313–327 (2011).

10. Kikinis, R., Pieper, S. D. & Vosburgh, K. G. 3D Slicer: A Platform for Subject-Specific Image Analysis, Visualization, and Clinical Support. in *Intraoperative Imaging and Image-Guided Therapy* 277–289 (Springer New York, New York, NY, 2014). doi:10.1007/978-1-4614-7657-3_19.

11. Fedorov, A. *et al.* 3D Slicer as an image computing platform for the Quantitative Imaging Network. *Magn Reson Imaging* 30, 1323–1341 (2012).
